# Impella 5.5 in left ventricular noncompaction syndrome as bridge to heart transplant

**DOI:** 10.1016/j.jhlto.2023.100051

**Published:** 2023-12-29

**Authors:** Shriya Sharma, Jose Ruiz, Pankaj Garg, Juan Leoni, Parag Patel, Jose Nativi, Melissa Lyle, Rohan Goswami

**Affiliations:** a Division of Advanced Heart Failure and Transplant, Mayo Clinic in Florida, Jacksonville, Florida; b Department of Cardiothoracic Surgery, Mayo Clinic in Florida, Jacksonville, Florida

**Keywords:** Impella 5.5, cardiogenic shock, transplant, LVAD, heart failure

## Abstract

**Background:**

Left ventricular non-compaction syndrome (LVNC) is an uncommon congenital disease characterized by prominent trabeculations and deep inter-trabecular recesses of the left ventricle wall. We present the first reported case of Impella 5.5 use in LVNC as a bridge to transplant in a highly sensitized patient.

**Methods:**

A 50-year-old female with a family history of sudden cardiac death, LVNC cardiomyopathy associated with TPM1 gene mutation, and recurrent hospitalizations due to heart failure was listed for heart transplantation. She had severely reduced systolic function (EF 20%) and high panel reactive antibodies (PRA). She was admitted for desensitization therapy. Hospitalized, she escalated to Dobutamine and Milrinone due to progressive shock. Impella 5.5 was inserted, and inotropes were reduced as her condition steadied. She tolerated desensitization and eventually underwent heart transplantation after 120 days of continuous Impella 5.5 support without developing hemolysis or pump thrombosis.

**Results:**

Post-transplant she was found to have concern for left sided stroke symtpoms but completely recovered and remians stable on follow up at 6 months. Due to the lack of prospective trials with large cohorts, managing patients with LVNC and cardiogenic shock is challenging. Novel approaches to complex patients with both Impella 5.5 support and desensitization therapies should be considered in the appropriate setting.

**Conclusion:**

Our case highlights the use of mechanical circulatory support in the management of cardiogenic shock within a small left ventricluar cavity in a sensitized patient. This approach should be considered in high-risk patients in whom durable left ventricular assist therapy is not feasible.

## Background

Left ventricular noncompaction syndrome (LVNC) is a relatively uncommon congenital heart disease characterized by excessively prominent trabeculations and deep intertrabecular recesses in the ventricle wall. There is considerable debate over whether the syndrome can also be acquired, although it is generally accepted that it is brought on by the intrauterine arrest of compaction of the cardiac fibers and meshwork, an essential phase in myocardial development. It is a primary genetic cardiomyopathy, according to the American Heart Association.^1^ Clinical symptoms might range from being asymptomatic to having debilitating congestive heart failure, arrhythmias, and systemic thromboemboli.^2^ In practice, smaller left ventricle (LV) cavity size is a high risk feature for the placement of durable support, such as Heart Mate 3 or heartmare LVAD, due to the risk of stroke, pump thrombosis, and arrhythmia.^3^ We present the first reported case of Impella 5.5 use in LVNC as a bridge to transplant in a complex sensitized patient with LVNC.

## Case presentation

A 50-year-old female blood group O positive with a family history of sudden cardiac death status post sub-cutaneous implantable cardioverter defibrillator and noncompaction cardiomyopathy associated with Tropomyosin 1 gene mutation and a history of recurrent hospitalizations due to shortness of breath and acute decompensated heart failure was listed for heart transplantation after evaluation was completed. She was inotrope dependent and had severely reduced systolic function with a left ventricular ejection fraction of 20%. While hospitalized she progressively declined and had escalation to Dobutamine 3 mcg/kg/min in addition to Milrinone 0.5 mcg/kg/min. Given limitations in medical therapy, worsening mixed venous saturation, and a Fick cardiac index of 2.0 liter/min/m^2^, it was felt that she required mechanical support placement as a bridge to heart transplantation. After a multidisciplinary review by cardiothoracic surgery, transplant cardiology, and critical care, she was not considered for durable let ventricular assist device (LVAD) due to small LV cavity size and sensitization, which would likely worsen with transfusion during surgery and limit future transplant potential. She underwent Impella 5.5 insertion through the right axillary artery. Inotropic agents were gradually reduced as the patient’s clinical and hemodynamic condition improved. Her hemodynamics remained stable while on Impella support for 120 days after which a suitable donor was identified. The patient exhibited human leukocyte antigen (HLA) Class 1 sensitivity of 96% and no sensitivity in HLA Class 2. Desensitization therapy before transplantation was completed with a self-limited transient ishemic attack-like event 1 month after but not clearly associated with desensitization.

Subsequent HLA monitoring revealed a significant reduction in antibodies, with no detectable HLA antibodies above prespecified thresholds (mean flouresence index >2 or 5,000) and a negative cytotoxic prospective crossmatch at the time a suitable donor was identified.

Plasmapheresis was also conducted before transplantation, thymoglobulin was administered intraoperatively, and pheresis was ongoing after the transplant for a total of 4 sessions. Concern for a post-transplant cerebrovascular event was noted in the perioperative period, but from an unclear etiology (i.e., bypass, peri-aortic clamp calcification, transient hypoxia, Impella explant, immunotherapy induction). She completely recovered and has been stable now for 6 months post-transplantation.

## Discussion

Our case presentation provides insight into complicated patients with advanced heart failure needs that can be successfully bridged and desensitized while supported on temporary mechanical circulatory support in the appropriate setting. Combining a specialized heart-team approach to personalize care for each patient can lead to acceptable outcomes and improvement in quality and quantity of life. Below we describe our Impella management for prolonged support, desensitization strategy, and surgical care at time of explantation.

### Brief review of LVNC

Noncompaction of ventricular myocardium was first described in association with other congenital anomalies, including coronary artery anomalies, complex cyanotic congenital heart disease, and obstruction of the right or left ventricular outflow tracts. In these situations, pressure overload or cardiac ischemia blocking regression of the embryonic myocardial sinusoids has been proposed as potential causes of the aberrant compaction process. As a result, deep intertrabecular recesses persist in communication with both the ventricular cavity and the coronary circulation.^2^ Alternatively, the pronounced hyper trabeculation could be due to altered regulation in cell proliferation, differentiation, and maturation during ventricular wall formation. Heart failure, arrhythmias, and embolic events have been identified as the main clinical manifestations of LVNC. Patients can have asymptomatic left ventricular dysfunction or have severe, incapacitating congestive heart failure, as in our patient.^4^

**Figure 1:**
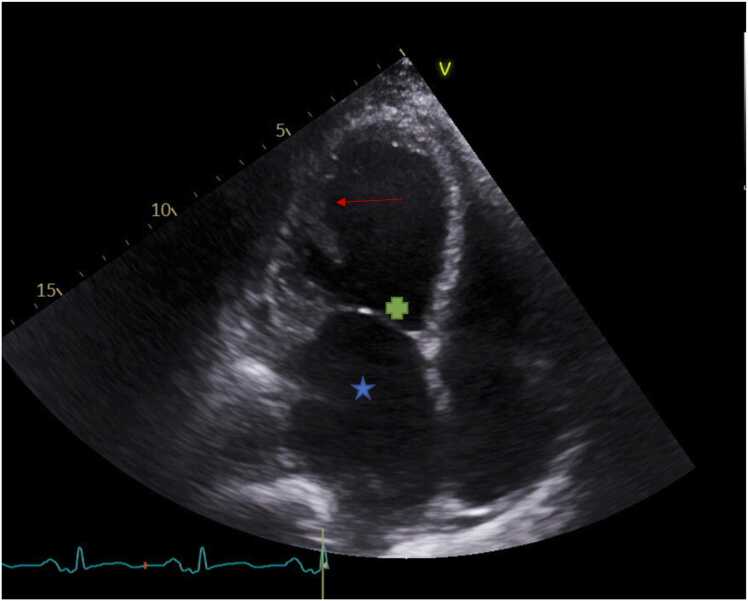
Apical 4-chamber view during transthoracic echocardiography. *Red arrow*: inside LV cavity pointing to crypts within LV-free wall; *: left atrium; +: mitral valve (closed). LV, left ventricle.

**Figure 2:**
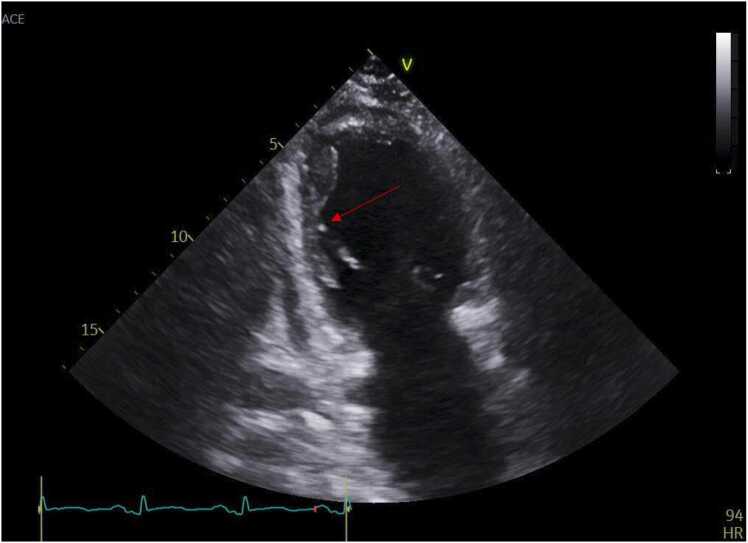
Apical 4-chamber view during transthoracic echocardiography. *Red arrow*: inside LV cavity with a regional increase in left ventricular trabeculation.

**Figure 3:**
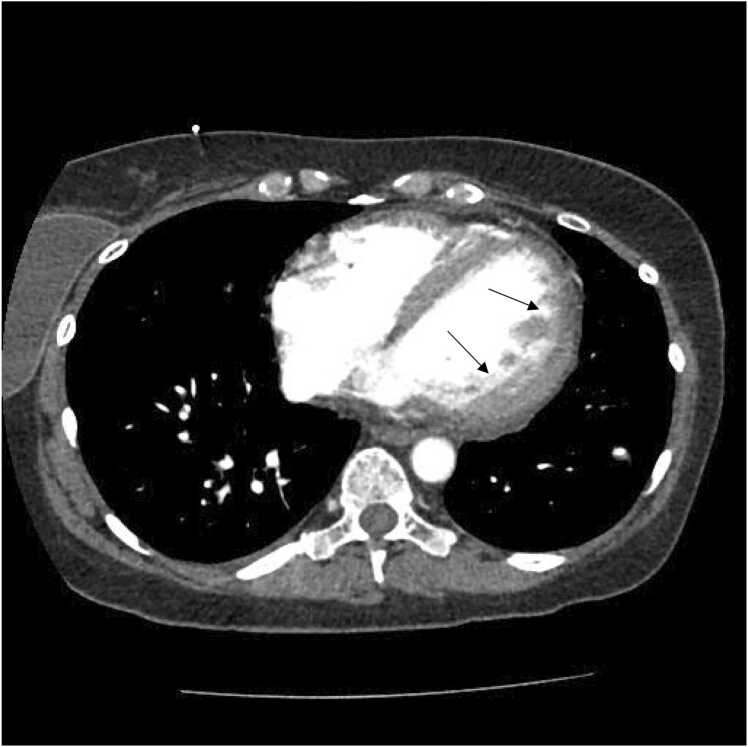
Contrast-enhanced axial CT scan showing hypertrophic ventricular trabeculations and deep interventricular recesses *(black arrows)*.

Several imaging modalities, including contrast ventriculography, computed tomography, 2D surface echocardiogram, and magnetic resonance imaging, have been employed to make the diagnosis even though echocardiography has traditionally been the test of choice for noncompaction. In situations where the quality of the echocardiographic image is poor, magnetic resonance imaging is helpful since it has a strong correlation with an echo for the localization and extent of noncompaction (Figure 1, Figure 2, Figure 3).^2^, ^5^ .

### Step-wise management of progressive heart failure cardiogenic shock

Heart failure cardiogenic shock (HFCS) is a life-threatening condition and temporary mechanical circulatory support (tMCS) might have a significant effect on the clinical outcome of patients with HFCS. Intra-aortic balloon pumps, Impella, and extracorporeal membrane oxygenation are a few examples of tMCS that have proven to be very effective in managing HFCS.

In the current era of HFCS management, one of the most used and promising tMCS devices is the Impella (Abiomed, Danvers). This percutaneous system uses a microaxial pump to provide left ventricular unloading and lower left ventricle end-diastolic pressure, reducing myocardial oxygen demand. Impella is designed to increase cardiac output while lowering end-diastolic ventricle pressure. Retrospective analyses from multicenter registries have recently revealed that Impella may improve patient survival in cardiogenic shock patients when adequate device selection, the timing of implant, as well as invasive hemodynamic monitoring, are provided. The Impella 5.5 has a peak flow rate of 6 liter/min and is designed for use in the management of HFCS.^6^, ^7^ The primary indication for using Impella 5.5 is the treatment of persistent cardiogenic shock brought on by acute myocardial infarction, cardiac surgery, or cardiomyopathy in those with isolated LV failure who do not respond to standard treatments (i.e., pressors, inotropes, and intra-aortic balloon pumps).^8^, ^9^ Impella is increasingly used as a bridging device for heart transplantation, a durable left ventricular assist device, or native heart recovery.^10^, ^11^

For some patients, durable LVAD implantation may be feasible and used as salvage or bridge to recovery or transplantation. Our case highlights the use of tMCS with Impella 5.5 in increasing the opportunity for highly sensitized patients with smaller LV cavities to remain supported and undergo desensitization therapy safely. Patients with smaller LV cavity size (<55-60 mm) due to underlying pathology (e.g., hypertrophy, genetic cardiomyopathy, infiltrative disorders) have clinically been considered at higher risk for durable device-related complications of stroke, thrombosis, or arrhythmia events in both axial and newer centrifugal configured LVAD designs.^12^, ^13^ Our group has published on the ability for optimization of end organ perfusion with tMCS Impella 5.5 support for renal recovery and pulmonary hypertension optimization, independent of this case.^14^, ^15^

Surgical placement of the device followed standard placement technique as follows: Following prepping and draping, a right axillary incision was performed. Heparin was administered. Activated clotting time was over 160. Axillary artery was explored and was turned around with vessel loops. A side biter clamp was placed on axillary artery. A 8 mm woven Vascutek graft was sewn onto the axillary artery with a running 5/0 polypropylene suture. Followed that under fluoroscopic guidance, a guidewire passed through the aortic valve and Impella 5.5 device was introduced to the axillary artery and was advanced into the left ventricular cavity. With the guidance of fluoroscopy and echocardiography, Impella 5.5 device was positioned and started with 4.0 liter flow. Axillary wound was closed in 2 layers with Vicryl sutures and a running suture on the skin.

Postimplant, we integrate factors such as mean arterial pressure, heart rate, and urine output with invasive hemodynamics, specifically, serum lactate, and SVO2 daily. Based upon these labs, we make changes to the device power level, inotrope dose, afterload reduction regimen, and diuresis goals as indicated to maintain sufficient cardiac support while optimizing right ventricle and LV filling pressures. Hemodynamic targets are a mean arterial pressure of 65 mm Hg, an right atrial pressure of 10 mm Hg, a pulmonary capillary wedge pressure <15 mm Hg, and a CI >2.2 liter/min/m^2^. Catheter position is assessed daily by insertion depth and in response to unexpected clinical changes or specific device alarms. An echocardiogram assesses the catheter position to identify the cannula relative to intracardiac structures. *The optimal cannula depth of the Impella 5.5 is 4.5* *cm* ±*0.5* *cm to the beginning of the inlet area. The device manufacturer suggests measuring catheter depth from the aortic annulus to the middle of the echolucent inlet area, (i.e., 0.5* *cm more than the distances stated above).* Anticoagulation was used to prevent clot formation on the motor/catheter, with the use of Bivalirudin at a concentration of 5 mg/ml in NaCl 0.9%, administered through a 50 ml infusion at a rate of 0.24 mg/kg/hour. Despite the recognized trend of device-related complications occurring more frequently with a longer duration of support our patient experienced intermittent atrial arrhythmias and few ventricular arrhythmias. We felt it prudent to utilize a background of low dose inotrope support to minimize the need for higher performance levels—thereby reducing the risk of suction into the ventricle and LV cavity irritation leading to arrhythmias or thrombosis. Our average performance level of the Impella device was P5, with a flow rate of 3.3 liter/min, and a purge pressure of 504 mm Hg.

Our institutional standard protocol was followed for intraoperative management of Impella removal. As such, the surgical team exposed the axillary insertion site prior to native heart explantation and cardiopulmonary bypass cannulation. She was cannulated on the ascending aorta and superior and inferior vena cava. The Impella device was removed with the native heart and retaining cord of the device was removed from the axillary wound.

### Desensitization and risk on tMCS

The patient was discussed for desensitization at our institutions multidisciplinary weekly selection meeting where she was felt to be a candidate while supported with Impella 5.5, having not shown a sustained response prior to Impella placement. Her inpatient desensitization protocol included a 3-day regimen repeated for 3 cycles prior to transplant. Pretransplant she underwent the following for 1 cycle: On day 1 and 2, she underwent therapeutic plasma exchange (PLEX) which consisted of plasma removal and replacement with ACD-A anticoagulant, NS and 5% albumin+fresh frozen plasma with sequential infusion of Bortezomib (1.3 mg/m^2^) with no therapy on day 3. Her second cycle of desensitization included 5 sessions of PLEX daily followed by intravenous immunoglobulin 2 mg/kg over 2 split doses. Her third cycle included 1 dose of Tocilizumab, with a planned second dose; however, a suitable donor was found and she underwent transplantation prior to the completion of this third cycle.

She did experience atrial arrhythmias during PLEX due to hypocalcemia and hypokalemia that responded to intermittent IV amiodarone bolus of 150 mg and were self limited to post-PLEX days. She had 1 episode of transient ishemic attack-like concern in the setting of ventricular tachycardia after her intravenous immunoglobulin and this was thought to be a serum reaction as all computed tomography heads and testing were negative and her symtpoms resolved in 24 hours.

Peritransplant offer management included a cytotoxic prospective crossmatch that showed T cell positivity—this was thought to be non-HLA interaction mediated and related to Tocilizumab that was given within 10 days of this offer being accepted. Our centers hematology pathologist felt that it was safe to proceed with the use of PLEX perioperatively. She underwent 1 session of PLEX prior to transplant and 4 sessions of PLEX after transplant while being induced with rabbit thymoglobulin 1.5 mg/kg for a total of 3 doses.

For the total duration of her desensitization therapy while supported on the Impella 5.5, she did not have any concerning findings based on daily lactate dehydrogenase, hemoglobin, and renal function panel/urinalysis for worsening hemolysis or progressive device-related thrombosis with purge pressure or flow monitoring. Furthermore, she was able to maintain ambulation and improve her functional capacity prior to organ transplant without the need for pump replacement or surgical repositioning. Minor bedside adjustments in the LV cavity to aortic outlet distance were made occasionally. She was on systemic bivalirudin with an activated partial thromboplastin time goal of 50 to 70 seconds and a bicarbonate purge solution for the Impella device.

### Post-transplant care

Our patient had a positive outcome despite the deviation from the initially planned desensitization protocol and the high risk of rejection. The patient utilized thymoglobulin as induction and due to pretransplant renal preservation she underwent early initiation of calcineurin inhibotor (tacrolimus, goal trough of 8-12) and standard antimetabolite therapy with mycophenolic acid at 1000 mg twice daily. She was placed on our post-transplant prednisone taper reaching 0.3 mg/kg at 3 months. Despite being considered high-risk due to a history of sensitization and elevated panel reactive antibodies, her endomyocardial biopsy showed grade 0, indicating an absence of cellular rejection, and C4D staining was also negative.

From a vascular event standpoint, she has fully recovered from her post-transplant stroke and is now functional and ambulating without assistive devices. She continues to follow in clinic and is now 6 months post-transplant without the formation of de novo donor-specific antibodies, renal replacement needs, or infectious complications.

## Conclusion

We highlight the safe use of Impella 5.5 support in a patient with LVNC as well as small LV cavity size with successful desensitization and transplantation after 120 days of support. This approach should be considered in high-risk patients in whom durable left ventricular assist therapy is not feasible.

## Disclosure statement

Dr Rohan Goswami is a speaker for abiomed. The other authors declare that they have no known competing financial interests or personal relationships that could have appeared to influence the work reported in this paper.

We would like to thank the Division of Heart Failure and Transplant, Jacksonville Florida.

The authors received no financial support for the research and publication of this article.
